# Single-cell RNA landscape of osteoimmune microenvironment in osteoporotic vertebral compression fracture and Kümmell's disease

**DOI:** 10.3389/fcell.2023.1276098

**Published:** 2023-12-15

**Authors:** Yude Xu, Suixiang Huang, Zhencong Li, Libing Dai, Hao Wu, Peigeng Wang, Xiguan Yao, Wei Luo, Yiming Liu, Weichao Yang, Yi Feng, Haixiong Miao, Jiake Xu, Dongping Ye

**Affiliations:** ^1^ Guangzhou Red Cross Hospital, Guangzhou Red Cross Hospital of Jinan University, Guangzhou, China; ^2^ Department of Pain Medicine, Guangzhou Red Cross Hospital, Guangzhou Red Cross Hospital of Jinan University, Guangzhou, China; ^3^ Department of Spinal Degeneration and Deformity Surgery, Affiliated Hospital of Guangdong Medical University, Zhanjiang, China; ^4^ The First Affiliated Hospital of Jinan University, Jinan University, Guangzhou, China; ^5^ School of Biomedical Sciences, The University of Western Australia, Perth, WA, Australia; ^6^ Shenzhen Institute of Advanced Technology, Chinese Academy of Sciences, Shenzhen, China

**Keywords:** osteoimmunology, osteoporosis, osteoporotic vertebral compression fracture, Kümmell’s disease, single-cell RNA sequencing

## Abstract

**Background:** Single-cell RNA sequencing (scRNA-seq) enables specific analysis of cell populations at single-cell resolution; however, there is still a lack of single-cell-level studies to characterize the dynamic and complex interactions between osteoporotic vertebral compression fractures (OVCFs) and Kümmell’s disease (KD) in the osteoimmune microenvironment. In this study, we used scRNA-seq analysis to investigate the osteoimmune microenvironment and cellular composition in OVCFs and KD.

**Methods:** ScRNA-seq was used to perform analysis of fractured vertebral bone tissues from one OVCF and one KD patients, and a total of 8,741 single cells were captured for single-cell transcriptomic analysis. The cellularity of human vertebral bone tissue was further analyzed using uniform manifold approximation and projection. Pseudo-time analysis and gene enrichment analysis revealed the biological function of cell fate and its counterparts. CellphoneDB was used to identify the interactions between bone cells and immune cells in the osteoimmune microenvironment of human vertebral bone tissue and their potential functions.

**Results:** A cellular profile of the osteoimmune microenvironment of human vertebral bone tissue was established, including mesenchymal stem cells (MSCs), pericytes, myofibroblasts, fibroblasts, chondrocytes, endothelial cells (ECs), granulocytes, monocytes, T cells, B cells, plasma cells, mast cells, and early erythrocytes. MSCs play an immunoregulatory function and mediate osteogenic differentiation and cell proliferation. The differentiation trajectory of osteoclasts in human vertebral bone tissue was also revealed. In addition, ECs actively participate in inflammatory infiltration and coupling with bone cells. T and B cells actively participate in regulating bone homeostasis. Finally, by identifying the interaction of ligand–receptor pairs, we found that immune cells and osteoclasts have bidirectional regulatory characteristics, have the effects of regulating bone resorption by osteoclasts and promoting bone formation, and are essential for bone homeostasis. It is also highlighted that CD8-TEM cells and osteoclasts might crosstalk via CD160–TNFRSF14 ligand–receptor interaction.

**Conclusion:** Our analysis reveals a differential landscape of molecular pathways, population composition, and cell–cell interactions during OVCF development into KD. OVCFs exhibit a higher osteogenic differentiation capacity, owing to abundant immune cells. Conversely, KD results in greater bone resorption than bone formation due to depletion of MSCs and a relatively suppressed immune system, and this immune imbalance eventually leads to vertebral avascular necrosis. The site of action between immune cells and osteoclasts is expected to be a new therapeutic target, and these results may accelerate mechanistic and functional studies of osteoimmune cell types and specific gene action in vertebral avascular necrosis and pathological bone loss diseases, paving the way for drug discovery.

## 1 Introduction

The World Health Organization identifies osteoporosis as one of the top 10 most serious diseases worldwide (Edidin et al., 2011), characterized by increased bone turnover and osteopenia with associated skeletal fragility, leading to an increased risk of fractures (Arceo-Mendoza and Camacho, 2021). The most common complication of osteoporosis is osteoporotic vertebral compression fracture (OVCF) (Tian et al., 2014), and its incidence is increasing year by year due to increased aging worldwide, affecting approximately 20% of the elderly population over 70 years of age and 16% of postmenopausal women worldwide (Karmakar et al., 2017). Kümmell’s disease (KD), known as avascular necrosis after OVCF (Lee et al., 2010), is also considered the terminal stage of OVCFs (Lim et al., 2018) and is a potential complication of up to one-third of OVCFs (Adamska et al., 2021). KD was first reported by Dr. Hermann Kümmell in 1895, defined as individuals who suffer from minor spinal trauma and are initially asymptomatic for weeks to months but subsequently develop symptomatic, progressive, and angulated kyphosis (Brower and Downey, 1981; Swartz and Fee, 2008). During the progression of KD, it can present with intractable pain and vertebral instability, leading to further collapse of the affected vertebral body, spinal canal stenosis, and neurological deficits (Xia et al., 2018). Most OVCF patients can be treated conservatively, and percutaneous vertebroplasty (PVP) and kyphoplasty (PKP) can achieve good results in the treatment of OVCF refractory to conservative treatment and KD without neurologic symptoms (Chen et al., 2021; Zhang et al., 2016). However, for patients with stage 3 KD combined with neurologic symptoms, internal fixation surgery and body reconstruction have a high incidence of complications (Chen et al., 2015), causing a heavy blow to the patient’s life and increasing the risk of disability, morbidity, and mortality (Xia et al., 2018).

During osteoporosis, bone resorption by osteoclasts outweighs bone formation by osteoblasts, and breaking of this balance leads to fragility fractures (Rodan and Martin, 2000). Various immune cells interact with osteoblasts and osteoclasts either by direct cell-to-cell contact or more likely through paracrine mechanisms (Fischer and Haffner-Luntzer, 2022). T helper cells (Th1, Th2, Treg, and Th17), B cells, dendritic cells (DCs), and macrophages actively participate in bone homeostasis (Dar et al., 2018). In 2000, the term “osteoimmunology” was coined by Arron and Choi (2000), an interdisciplinary field of research that combines existing fields of osteobiology and immunology. Since then, increasing attention has been paid to the interaction between immune cells and bone cells, especially in inflammatory bone diseases such as osteoporosis, rheumatoid arthritis, and periodontal disease, along with conditions like Paget’s disease of bone, primary bone cancers, and skeletal complications associated with bone metastases from breast and prostate tumors (Dar et al., 2018; Rodan and Martin, 2000). Post-traumatic osteonecrosis is the most recognized pathogenetic hypothesis for KD (Gou et al., 2023). Following OVCFs, how KD develops vertebral avascular necrosis and what happens to its osteoimmune microenvironment remain poorly understood. Therefore, with the arrival of a new era in the field of osteoimmunology research, it will bring a new theoretical basis for the prevention and treatment of diseases associated with pathological bone loss under inflammatory conditions.

Single-cell RNA sequencing (scRNA-seq) enables the exploration of heterogeneity and cell–cell communication in complex tissues at high resolution. It also provides a broader characterization of transcriptome profiles with higher resolution and accuracy (Wang et al., 2022). This technique allows a more specific understanding of the interactions between cells involved in pathological bone loss and immune cells (Chen et al., 2022). The aim of this study was to analyze the osteoimmune microenvironment and cellular composition involved in vertebral bone tissue samples in a patient with OCVF and a patient with KD by scRNA-seq. Thus, it reveals how the interaction between bone cells and immune cells is involved in vertebral ischemic osteonecrosis and pathological bone loss in OCVFs and KD.

## 2 Materials and methods

### 2.1 Patient and sample collection

Bone tissue samples were collected from two human vertebral fractures, and OCVF bone tissue samples were obtained from an 83-year-old male patient who underwent “percutaneous lumbar 4 vertebral balloon angioplasty” at the Department of Orthopaedics, Guangzhou Red Cross Hospital, Jinan University on 20 June 2022. The patient came to our hospital for surgical treatment due to lower back pain caused by acute fall trauma history. Preoperative lumbar MR revealed an acute compression fracture of the L4 vertebral body (bone marrow edema), but no intravertebral vacuum sign (IVC sign) was found in the fractured vertebral body. According to the WHO diagnostic criteria for osteoporosis, the lowest bone mineral density (BMD) measurement (left femoral neck) T value of this patient was 2.8, which meets the criteria for the diagnosis of osteoporosis, and the patient’s diagnosis was consistent with acute OVCF.

KD fractured vertebral bone tissue samples were obtained from a 73-year-old female patient who underwent “L2 vertebroplasty + percutaneous L1 and L3 pedicle screw internal fixation” at the Department of Orthopaedics, Guangzhou Red Cross Hospital, Jinan University on 14 June 2022. The patient had a history of falls 4 months ago and experienced symptomatic relief of lower back pain several days later, followed by an asymptomatic period, and came to our hospital 1 week before admission for surgical treatment due to severe lower back pain. Preoperative lumbar MR showed a compression fracture of the L2 vertebral body with a vacuum sign of the vertebral body in the fractured vertebral body. According to the WHO diagnostic criteria for osteoporosis, the lowest BMD measurement (lumbar spine) T value in this patient was −3.0, which meets the criteria for the diagnosis of osteoporosis, and the patient’s diagnosis was consistent with KD. Basic data, relevant clinical data, and tissue samples were collected from the above two patients after signing the informed consent form.

### 2.2 Preparation of the single-cell suspension

The tissues were surgically removed and kept in MACS Tissue Storage Solution (Miltenyi Biotec) until processing. The tissue samples were processed as described below. In brief, the samples were first washed with phosphate-buffered saline (PBS), minced into small pieces (approximately 1 mm^3^) on ice, and enzymatically digested with 3-mg/mL collagenase I (Worthington), 6-mg/mL collagenase II (Worthington), 4-mg/mL dispase II (Sigma), and 10% FBS in DMEM for 70 min at 37°C, with agitation. After digestion, the samples were sieved through a 70-μm cell strainer and centrifuged at 300 g for 5 min. After the supernatant was removed, the pelleted cells were suspended in red blood cell lysis buffer (Miltenyi Biotec) to lyse red blood cells. After washing with PBS containing 0.04% BSA, the cell pellets were re-suspended in PBS containing 0.04% BSA and re-filtered through a 35-μm cell strainer. Dissociated single cells were then stained with Am/Draq7 for viability assessment using a BD Scanner Cell Analyzer.

### 2.3 Preparation of single-cell suspensions for library construction and scRNA sequencing

A BD Rhapsody analysis system was used to prepare single-cell whole transcriptome. The cell suspension was loaded into the BD Rhapsody cartridge containing a microwell array with>220,000 partitions. Single-cell capture was achieved by random distribution of single cells that settled into the microwells by gravity, followed by the addition of beads with oligonucleotide barcodes for pairing with the cells. After cell lysing, the barcoded beads containing the captured mRNAs were retrieved, washed, and then, subjected to reverse transcription and exonuclease I digestion. Single-cell transcriptomic sequencing libraries were prepared by following a series of PCR steps including random priming and extension (RPE), RPE PCR, and whole-transcriptome amplification (WTA) index PCR according to the manufacture’s instruction. Normalized libraries were sequenced on NovaSeq Illumina with a 150 bp paired-end run.

### 2.4 Processing of scRNA sequencing data

scRNA-seq data analysis was performed by NovelBio Bio-Pharm Technology Co., Ltd., with the NovelBrain Cloud Analysis Platform. We applied fastp (Chen et al., 2018) with default parameter filtering the adaptor sequence and removed the low-quality reads to achieve clean data. UMI-tools (Smith et al., 2017) was applied for single-cell transcriptome analysis to identify the cell barcode whitelist. The UMI-based clean data were mapped to the human genome (GRCh38 Ensemble: version 104) utilizing STAR (Dobin et al., 2013) mapping with a customized parameter from the UMI-tools standard pipeline to obtain the UMI counts of each sample. Cells containing over 200 expressed genes and mitochondria UMI rate below 20% passed the cell quality filtering, and mitochondria genes were removed in the expression table.

### 2.5 Cell clustering analysis, visualization, and annotation

The Seurat package (version: 4.0.3, https://satijalab.org/seurat/) was used for cell normalization and regression based on the expression table according to the UMI counts of each sample and percent of mitochondria rate to obtain the scaled data. PCA was constructed based on the scaled data with the top 2,000 high variable genes, and the top 10 principals were used for tSNE construction and UMAP construction. The fastMNN function (k = 5, d = 50, approximate = TRUE) in R package scran (v1.12.1) was used to apply the mutual nearest-neighbor method to correct for batch effect among the samples.

Utilizing the graph-based cluster method (resolution = 0.8), we acquired the unsupervised cell cluster result based on the PCA top 10 principals, and we calculated the marker genes by the FindAllMarkers function with the Wilcoxon rank sum test algorithm under the following criteria: 1. lnFC >0.25; 2. *p* value<0.05; 3. min.pct>0.1. In order to identify the cell type detailed, the clusters of the same cell type were selected for re-tSNE analysis, graph-based clustering, and marker analysis.

### 2.6 Pseudo-time analysis

We applied the single-cell trajectory analysis utilizing Monocle 2 (http://cole-trapnell-lab.github.io/monocle-release) using DDR-Tree and the default parameter. Before Monocle analysis, we selected marker genes of the Seurat clustering result and raw expression counts of the cell passed filtering. Based on the pseudo-time analysis, branch expression analysis modeling (BEAM analysis) was applied for branch fate-determined gene analysis.

### 2.7 Cell communication analysis

To enable a systematic analysis of cell–cell communication molecules, we applied cell communication analysis based on the Cellphone DB (Vento-Tormo et al., 2018), a public repository of ligands, receptors, and their interactions. The membrane, secreted, and peripheral proteins of the cluster of different time points were annotated. Significant mean and cell communication significance (*p*-value<0.05) were calculated based on the interaction and the normalized cell matrix achieved by Seurat Normalization.

### 2.8 SCENIC analysis

To assess transcription factor regulation strength, we applied the Single-Cell rEgulatory Network Inference and Clustering (pySCENIC, v0.9.5) (Aibar et al., 2017) workflow, using the 20-thousand motifs database for RcisTarget and GRNboost.

#### 2.8.1 QuSAGE analysis (gene enrichment analysis)

To characterize the relative activation of a given gene set such as pathway activation, we performed QuSAGE (Yaari et al., 2013) (2.16.1) analysis.

#### 2.8.2 Differential gene expression analysis

To identify differentially expressed genes among the samples, the function FindMarkers with the Wilcoxon rank sum test algorithm were used under the following criteria: 1) lnFC >0.25; 2) *p* value<0.05; and 3) min.pct>0.1.

#### 2.8.3 Co-regulated gene analysis

To discover the gene co-regulation network, the find_gene_modules function of Monocle 3 (Cao et al., 2019) was used with the default parameters.

#### 2.8.4 Gene Ontology analysis

Gene Ontology (GO) analysis was performed to facilitate elucidation of the biological implications of marker genes and differentially expressed genes. We downloaded the GO annotations from NCBI (http://www.ncbi.nlm.nih.gov/), UniProt (http://www.uniprot.org/), and Gene Ontology databases (http://www.geneontology.org/). Fisher’s exact test was applied to identify the significant GO categories, and FDR was used to correct the *p*-values.

#### 2.8.5 Pathway analysis

Pathway analysis was used to find out the significant pathway of the marker genes and differentially expressed genes according to the KEGG database. We turned to Fisher’s exact test to select the significant pathway, and the threshold of significance was defined by *p*-value and FDR.

### 2.9 Statistical analysis

All statistical analyses and figures were generated using R software (version 3.6.3). A *p*-value <0.05 was considered statistically significant.

## 3 Results

### 3.1 ScRNA-seq and cellular composition of human vertebral bone tissue

**FIGURE 1:**
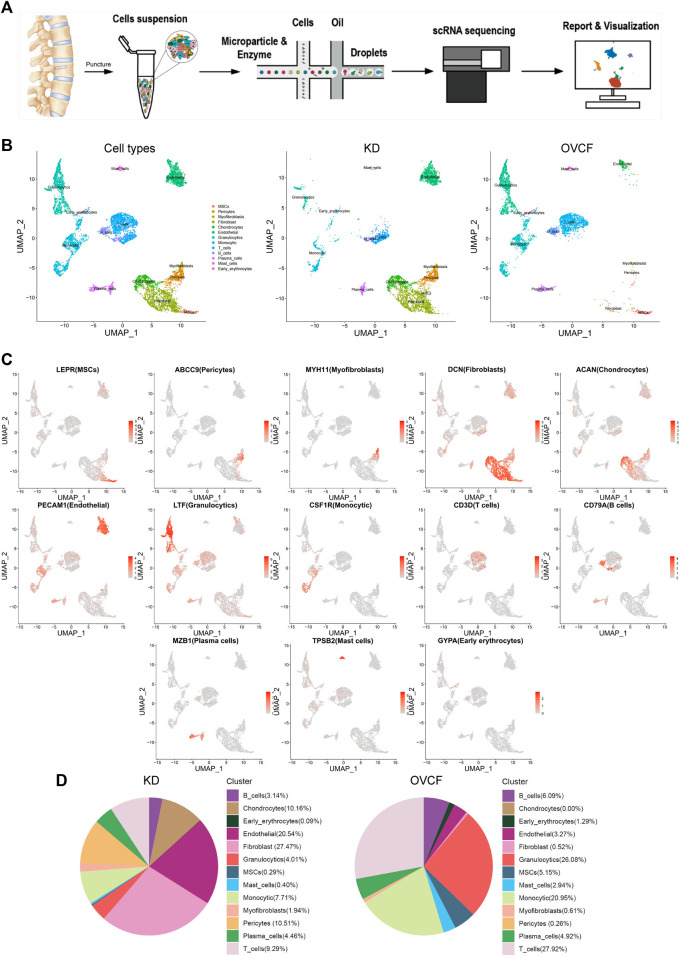
Overview of 8,741 single cells from human vertebral bone tissue from KD and OVCF samples. **(A)** Study overview. **(B)** Based on uniform manifold approximation and projection (UMAP), dimensionality reduction visualization is performed and coloring is marked according to the cell type. From left to right are 8,741 cells of KD and OVCF samples, 4,489 cells of the KD sample, and 4,252 cells of the OVCF sample. **(C)** UMAPs are shown in Figure 1B, showing differentially highly expressed classical marker genes for each cell type and coloring according to their expression. **(D)** Proportional pie chart for each cell type in the respective sample.

To initially explore the cellular composition in human vertebral bone tissues, we performed scRNA-seq analysis of fractured vertebral bone tissues from one OVCF patient and one KD patient (Figure 1A). Clinical sample data are presented in Section 2. After standard data processing and quality filtering, batch correction between the samples was performed by the mutual nearest-neighbor (MNN) algorithm (Section 2). We captured a total of 8,741 single cells for single-cell transcriptomic analysis, including 4,252 cells from the OVCF samples and 4,489 cells from the KD samples. Based on uniform manifold approximation and projection (UMAP), 13 cell populations (two-sample combined and sub-sample display) were preliminarily obtained (Figure 1B).

Next, we identified each cell population according to their differentially highly expressed classical marker genes and preliminarily classified and annotated these 13 cell populations, which were LEPR + MSCs, ABCC9 + pericytes, MYH11 + myofibroblasts, DCN + fibroblasts, ACAN + chondrocytes, PECAM1 + endothelial cells, LTF + granulocytics, CSF1R + monocytic, CD3D + T cells, CD79A+ B cells, MZB1 + plasma cells, TPSB2 + mast cells, and GYPA + early erythrocytes (Figure 1C).

We compared the proportion of each cell population in the two samples. Notably, pericytes, fibroblasts, chondrocytes, and endothelial cells (ECs) were present in a higher proportion of the KD samples, but mesenchymal stem cells (MSCs) were present in only 0.29%. The proportion of immune cells (T cells, B cells, monocytes, mast cells, and granulocytes) was significantly reduced compared with OVCFs (Figure 1D). This is similar to the work of Avin et al. (2022) who reported that scRNA-seq accounted for five intramedullary canal tissue samples obtained by reaming at the time of surgical repair of nonunion of femoral fractures and four types of cells from autologous bone controls collected at the time of autologous bone graft harvest. According to the above results, we tentatively speculated that OVCFs had higher osteogenic differentiation ability and more enriched interaction with immune cells. Conversely, immune dysregulation leads to greater bone resorption than bone formation in the KD samples due to the depletion of MSCs and a relatively suppressed state with the immune system, ultimately leading to vertebral avascular osteonecrosis.

### 3.2 MSCs exert immunomodulatory functions and mediate osteogenic differentiation and cell proliferation

**FIGURE 2:**
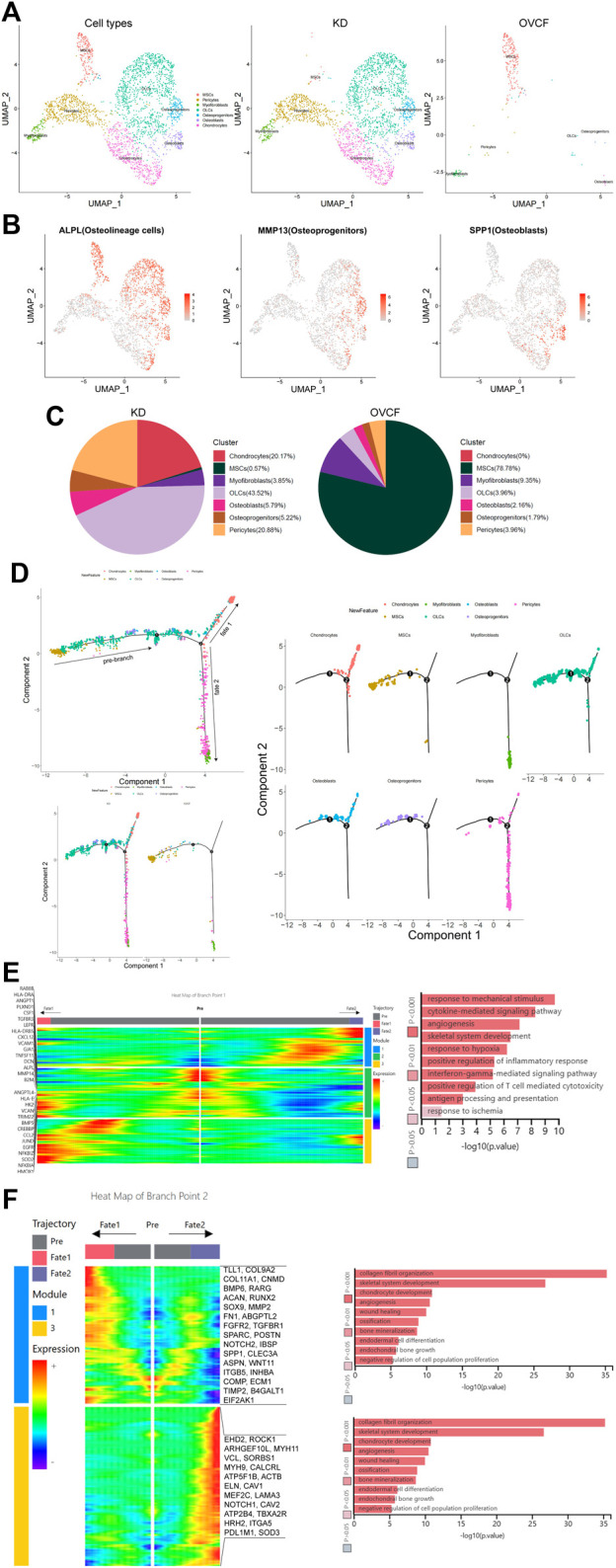
Distinct subclusters derived from MSC differentiation. **(A)** All preliminary groups of MSCs, pericytes, myofibroblasts, fibroblasts, and chondrocytes were combined to reconstruct the UMAP profile and labeled for staining according to the cell type. From left to right are the KD and OVCF samples, the KD sample, and the OVCF sample. **(B)** UMAPs are shown in Figure 2A and show differentially highly expressed classical marker genes in the distinct cell cluster of fibroblasts and colored according to their expression. **(C)** Proportional pie chart of subclusters of cells derived from the differentiation of MSCs. **(D)** The upper left panel shows the analysis of single-cell trajectories of all MSCs, OLCs, osteoprogenitor cells, osteoblasts, chondrocytes, pericytes, and myofibroblasts in the KD and OVCF samples; the lower left panel shows the differentiation trajectories of the above cells in KD versus OVCF; and the right panel shows the single-cell trajectories of each cell type. **(E)** The left panel is a heatmap of branch point 1 shown in Figure 2D, with the GO terms enriched by the pre-branch on the right side of the heatmap and the genes differentially highly expressed based on the GO terms enriched by the pre-branch on the left side of the heatmap. **(F)** The left panel is a heatmap of branch point 2 shown in Figure 2D, the right panel is the GO terms enriched in cell fate 1 and cell fate 2, and the right side of the heatmap is the genes differentially highly expressed based on the GO terms enriched in cell fate 1 and cell fate 2.

After initial clustering, we then subdivided and identified MSCs, pericytes, myofibroblasts, fibroblasts, and chondrocytes, of which we identified three subsets in fibroblasts, which were ALPL + osteolineage cells (OLCs), MMP13 + osteoprogenitors, and SPP1 + osteoblasts (Figures 2A, B).

MSCs are multipotent stromal cells, which mainly include exerting immunoregulatory functions and osteogenic differentiation ability, have the ability to regulate cell proliferation and adipogenic differentiation, and have a wide range of application potential in the treatment of diseases with tissue regeneration (Wang et al., 2021; Xie et al., 2022). Given that MSCs accounted for up to 78.78% of OVCFs, osteolineage cells, osteoprogenitors, osteoblasts, and chondrocytes accounted for a higher proportion of the KD samples than the OVCF samples. Pericytes were also significantly elevated in the KD samples compared with the OVCF samples. Although the proportion of myofibroblasts was lower in the KD samples than that in OVCFs, the number of cells was as much as 3-fold higher (Figure 2C).

To further investigate the differentiation trajectory of human vertebral bone tissues originating from MSCs, we used pseudo-time analysis to mimic the specific differentiation process of MSCs. Bounded by node 1, we could observe that MSCs mainly accumulated in the pre-branch and gradually differentiated toward OLCs, osteoprogenitors, osteoblasts, chondrocytes, pericytes, and myofibroblasts. Bounded by node 2, pseudo-time analysis started with MSCs, which then divided into two main branches, cell fate 1 differentiated toward OLCs, osteoblasts, and chondrocytes, while cell fate 2 differentiated into pericytes and myofibroblasts (Figure 2D).

In order to investigate the main biological process of its branches, we performed GO analysis of the pre-branch bounded by node 1, and we observed the following: 1) response to mechanical stimulation (DCN, CXCL12, MMP14, and JUND); 2) cytokine-mediated signaling pathway (HMOX1, CCL2, LEPR, TNFRSF11, and CSF1); 3) angiogenesis (ANGPT1, ANGPTL4, and PLXND1); 4) skeletal system development (ALPL, VCAN, and BMP5); 5) response to hypoxia (CREBBP, TGFBR3, and SOD2); 6) positive regulation of inflammatory response (NFKBIA, NFKBIZ, and EGFR); 7) interferon-γ-mediated signaling pathway (VCAM1, HLA-DRA, B2M, HLA-DR5, and TRIM22); 8) positive regulation of T-cell-mediated cytotoxicity (B2M, HLA-DRA, HLA-and DRE); 9) antigen processing and presentation (HLA-DRB5 and RAB8B); and 10) response to ischemia (HK2, GJA1, and CSF1) (Figure 2E).

Bounded by node 2, we proceeded to investigate the biological process of cell fate 1 and cell fate 2. We observed the following GO terms enriched in cell fate 1: 1) collagen fibril organization (TLL1, COL9A2, and COL11A1); 2) skeletal system development (TLL1, COL9A2, CNMD, and BMP6); 3) chondrocyte development (RARG, ACAN, RUNX2, SOX9, and COL11A1); 4) angiogenesis (MMP2, FN1, and ABGPTL2); 5) wound healing (FGFR2, TGFBR1, SPARC, FN1, POSTN, and NOTCH2); 6) ossification (IBSP, COL11A1, SPP1, BMP6, and CLEC3A); 7) bone mineralization (ASPN, FGFR2, WNT11, and IBSP); 8) endodermal cell differentiation (ITGB5, MMP2, FN1, and INHBA); 9) endochondral bone growth (FGFR2, COMP, and ECM1); and 10) negative regulation of cell population proliferation (TIMP2, B41, and EIF2GAL1) (Figure 2F).

Bounded by node 2, in cell fate 2, most cells are pericytes and myofibroblasts and only a small proportion is chondrocytes, while OLCs, osteoprogenitors, and osteoblasts cannot pass through node 2, and GO terms enriched in cell fate 2 are as follows: 1) actin cytoskeleton organization (EHD2, ROCK1, and ARHGEF10L); 2) muscle contraction (MYH11, VCL, and SORBS1); 3) angiogenesis (MYH9, CALCRL, and ATP5F1B); 4) platelet aggregation (VCL, ACTB, and MYH9); 5) skeletal muscle tissue development (ELN, CAV1, and MEF2C); 6) regulation of cell migration (LAMA3, VCL, ROCK1, and NOTCH1); 7) regulation of cytosolic calcium ion concentration (CAV2, CAV1, and ATP2B4); 8) positive regulation of vasoconstriction (TBXA2R, CAV1, and HRH2); 9) negative regulation of anoikis (CAV1, NOTCH1, and ITGA5); and 10) response to hypoxia (CAV1, PDL1M1, and SOD3) (Figure 2F).

Overall, based on GO analysis of the pre-branch of node 1 and the two main branches of node 2, we speculate that the causes of pathological bone loss and vertebral avascular osteonecrosis may be due to the body’s response to mechanical stimulation, ischemia, and hypoxia after trauma, as well as the activation of T cells, thereby promoting the combined effects of T-cell-mediated cytotoxicity and interferon-γ-mediated signaling pathways. More importantly, based on the two branches of node 2, we believe that MSCs have multipotent properties such as regulating immunity, inflammatory response promoting osteogenesis, blood vessels, cartilage, and muscle differentiation and development, and other biological processes such as cell proliferation.

### 3.3 Osteoimmune microenvironment and osteoclast differentiation trajectory in the monocytic cell lineage

**FIGURE 3:**
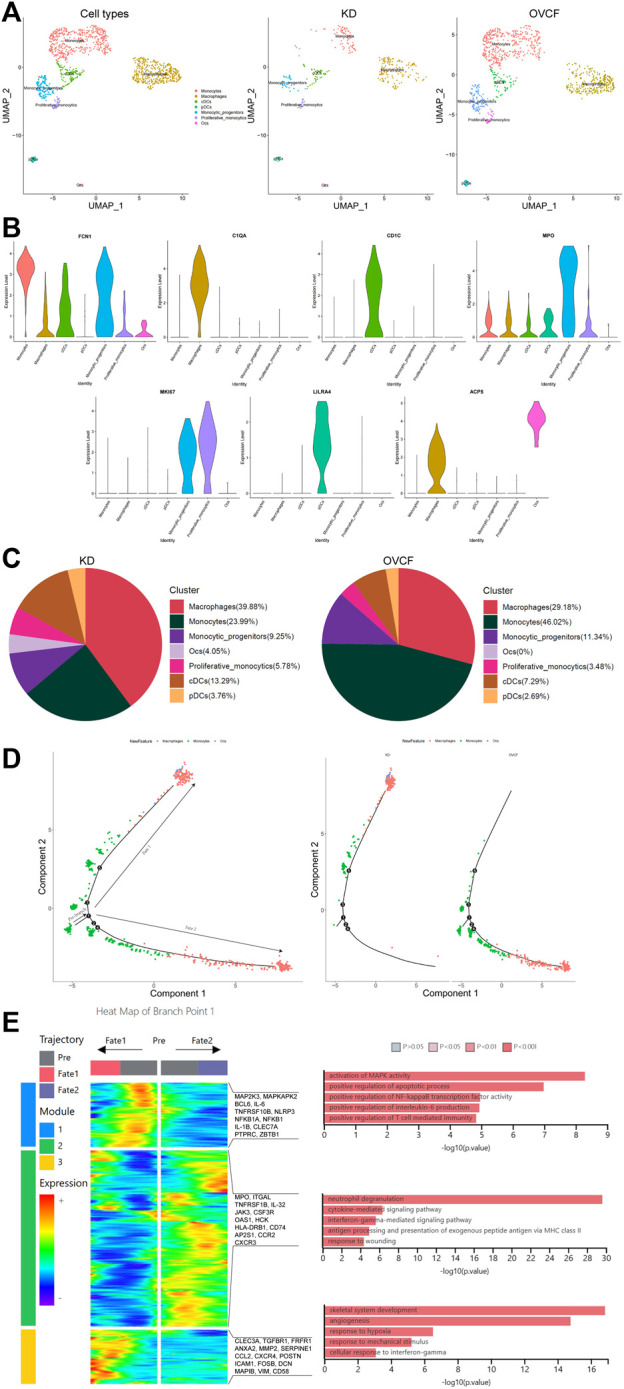
Differentiation track of osteoclasts in vertebral bone tissue. **(A)** Preliminary clusters of monocyte lineages were merged to reconstruct UMAP profiles, with coloration labeled by the cell type. From left to right are KD and OVCF samples, the KD sample, and the OVCF sample. **(B)** Violin plots of marker genes for monocyte lineage cell subsets. **(C)** Proportional pie chart of monocyte lineage cell subsets in respective samples. **(D)** The left panel shows the analysis of the single-cell trajectories of all monocytes, macrophages, and osteoclasts in KD and OVCF samples; the right panel shows the single-cell trajectories of the above three cells in divided samples. **(E)** The left panel is a heatmap of branch point 1 shown in Figure 3D, the right panel is based on the GO terms of the three modules, and the right side of the heatmap is the genes with differentially high expression of the GO terms corresponding to each of the three modules.

After initial clustering, we then combined two samples of monocyte lineage cells for further subdivision and identification, and seven cell types were preliminarily identified based on differentially highly expressed classical marker genes, which were FCN1 + monocytes, C1QA + macrophages, CD1C + conventional dendritic cells (cDCs), MPO + monocytic_progenitors, MKI67 + proliferative monocytics, LILRA4 + plasmacytoid dendritic cells (pDCs), and ACP5 + osteoclasts (Ocs) (Figures 3A, B).

We found a slightly higher proportion of conventional dendritic cells, plasmacytoid dendritic cells, and proliferative monocytics in the KD samples compared to OVCFs, whereas the proportion of monocyte progenitors was slightly lower than that in the OVCF samples. Interestingly, the proportion of monocytes was twice as high in the OVCF sample as in KD, but the proportion of macrophages was higher in the KD samples than that in the OVCF samples, and osteoclasts (4.05% of total cells) were found only in the KD samples but not in the OVCF samples (Figure 3C).

To validate osteoclast differentiation trajectories in human vertebral bone tissues, we extracted monocytes, macrophages, and osteoclasts from two samples for pseudo-time analysis. Bounded by node 1, we observed monocytes differentiating in two distinct directions. During differentiation to cell fate 1, monocytes gradually differentiate into macrophages and osteoclasts, while during differentiation to cell fate 2, monocytes differentiate into macrophages and have not differentiated into osteoclasts. More heavily, cells of cell fate 1 were mostly concentrated in the KD samples, whereas cells of cell fate 2 were almost concentrated in the OVCF samples (Figure 3D).

To investigate the biological process of the two main branches of monocyte differentiation (bounded by node 1), GO analysis was then performed on their enriched genes. We can observe that in the heatmap, module 1 depicts the process of monocyte differentiation into macrophages, which we call pre-osteoclast formation; module 3 depicts the period of macrophage differentiation into osteoclasts, so as to understand the trajectory of osteoclast differentiation in the KD samples as well as their biological process. Module 2, on the other hand, depicts the process of monocyte differentiation to cell fate 2, which represents the biological process of monocyte differentiation in the OVCF samples (Figure 3D).

During the progression of KD, module 1 enriched GO terms that differentiate monocytes into osteoclastic precursor cells and these include 1) activating MAPK activity (MAP2K3, MAPKAPK2, and IL-1B); 2) positive regulation of the apoptotic process (BCL6, IL-6, and TNFRSF10B); 3) positive regulation of NF-kappaB transcription factor activity (NLRP3, NFKB1A, and NFKB1); 4) positive regulation of interleukin-6 production (IL-6, IL-1B, and CLEC7A); and 5) positive regulation of T-cell-mediated immunity (PTPRC, IL-1B, and ZBTB1). Based on enriched GO terms and highly expressed genes, we suggest that the activation of MAPK and NF-kappaB signaling pathways by monocytes/macrophages and the promotion of apoptotic processes, interleukin-6 production, and T-cell-mediated immune responses may play an important role in regulating osteoclast formation. Numerous studies have reported that MAPK, the NF-kappaB signaling pathway (Liang et al., 2022), and interleukin-6 production (Yoshitake et al., 2008) play a key role in regulating osteoclast formation (Figure 3E).

On the trajectory of final differentiation into osteoclasts, GO terms enriched in module 3 were as follows: 1) skeletal system development (CLEC3A, TGFBR1, and FRFR1); 2) angiogenesis (ANXA2, MMP2, SERPINE1, and CCL2); 3) response to hypoxia (CXCR4, POSTN, and ICAM1); 4) response to mechanical stimulus (FOSB, DCN, MAPIB, and POSTN); and 5) cell response to interferon-γ (CCL2, VIM, and CD58). Among these, the GO term “skeletal system development” reflects that osteoclasts also promote osteogenic differentiation during differentiation, demonstrating that osteoclasts may have a coupling mechanism for osteoblasts and revealing the bidirectional regulation of osteogenic lineage cells and osteoclasts to maintain bone homeostasis. However, the GO term angiogenesis enriched gene ANXA2 has the function of enhancing osteoclast formation and bone resorption, while MMP2 has the function of reducing bone mineralization and promoting osteolysis (Li et al., 2021); most intuitively, the combined effect of the body’s continuous response to hypoxia and mechanical stimulation may ultimately make osteoclast bone resorption greater than osteoblast bone formation, which in turn leads to pathological bone loss and vertebral ischemic osteonecrosis, further demonstrating the altered differentiation trajectory of monocyte–macrophage–osteoclast in human vertebral bone tissue (Figure 3E).

During the progression of OVCFs (cell fate 2), GO terms enriched in module 2 were as follows: 1) neutrophil degranulation (MPO, ITGAL, and TNFRSF1B); 2) cytokine-mediated signaling pathway (IL-32, JAK3, and CSF3R); 3) interferon-γ-mediated signaling pathway (OAS1, HCK, and HLA-DRB1); 4) antigen processing and presentation of exogenous peptide antigen via MHC class II (CD74, HLA-DRB1, and AP2S1); and 5) response to wounding (CCR2 and CXCR3). Because the body activates inflammatory response processes such as “neutrophil degranulation,” “the cytokine-mediated signaling pathway,” “the interferon-γ-mediated signaling pathway,” “antigen processing and presentation of an exogenous peptide antigen via MHC class II,” and “response to wounding” after acute trauma, it then reveals that immune cells actively participate in regulating bone homeostasis and reveal the heterogeneity of their monocytes (Figure 3E).

### 3.4 Endothelial cells actively participate in inflammatory infiltration and coupling with osteopoiesis

**FIGURE 4:**
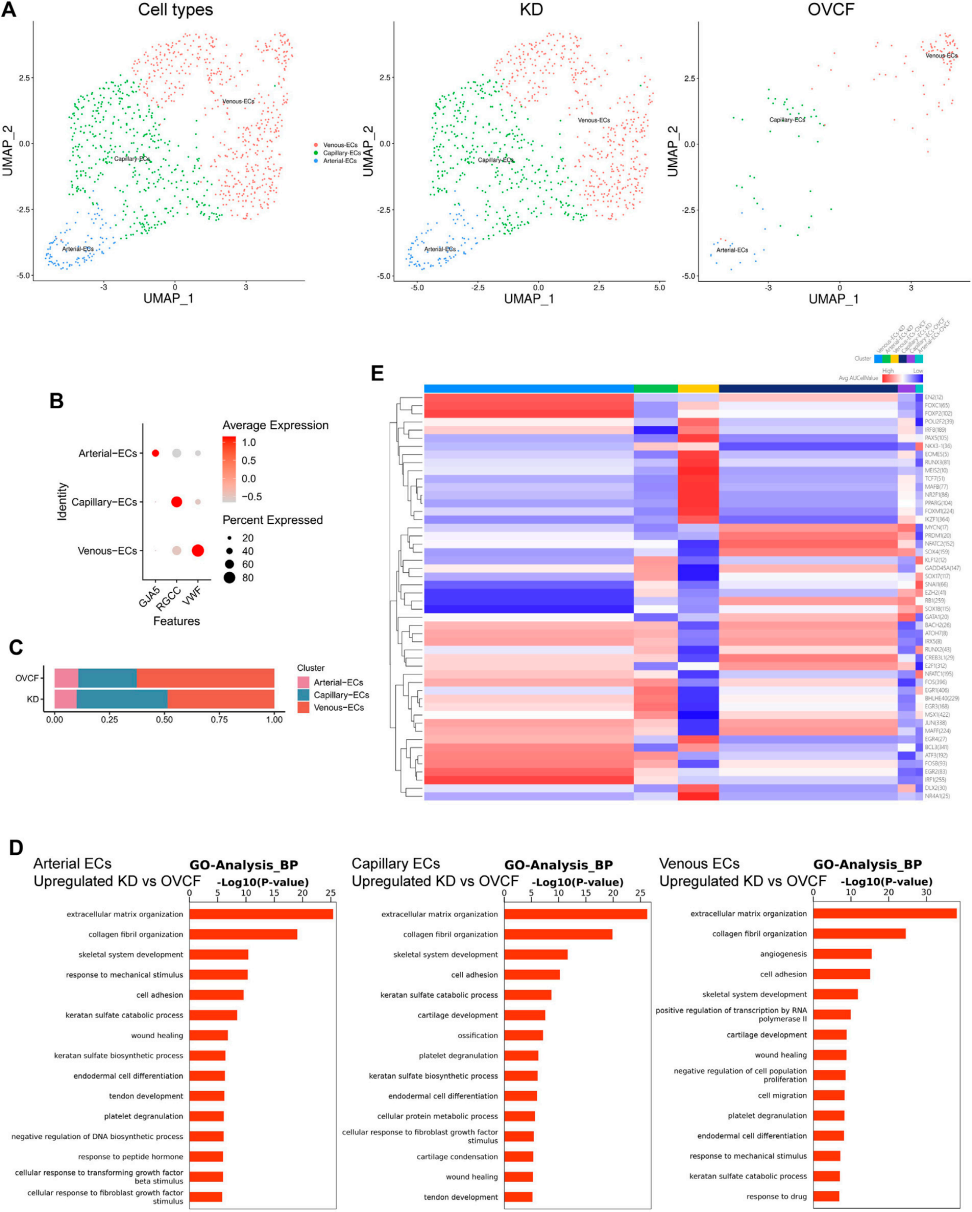
Endothelial cells actively participate in inflammatory infiltration and coupling with osteopoiesis. **(A)** All endothelial cells initially grouped were pooled to reconstruct the UMAP profile, with coloration labeled by the cell type. From left to right are the KD and OVCF samples, the KD sample, and the OVCF sample. **(B)** Bubble plots of marker genes for the endothelial cell subcluster. **(C)** Bar chart of the endothelial cell subcluster in respective sample proportions. **(D)** Biological process of GO enrichment was performed for genes differentially upregulated in endothelial cells in KD samples compared with OVCF samples. The left panel shows arterial endothelial cells; the middle panel shows capillary endothelial cells; and the right panel shows venous endothelial cells. **(E)** SCENIC was used to analyze the heatmap of the number and intensity of genes regulated by each transcription factor in each of the three endothelial cell subsets in the two samples, with red representing stronger regulation and blue representing weaker regulation.

Based on the preliminary cluster EC family, we combined the ECs of the two samples for further subdivision and identification, preliminarily identified three cell species based on the differentially highly expressed classical marker genes, which were GJA5 + arterial ECs, RGCC + capillary ECs, and VWF + venous ECs, and constructed two-sample and sub-sample UMAP maps according to the cell type (Figures 4A, B). We found that the number of ECs was much higher in the KD samples than in the OVCF samples, and abundant ECs may be involved in active inflammatory infiltration, with venous ECs and capillary ECs accounting for up to 48.5% and 41.5%, respectively, in the KD samples, while arterial ECs accounted for only 10%. However, venous ECs were equally represented in 62.6% of the OVCF samples, and capillary ECs and arterial ECs accounted for 26.6% and 10.8%, respectively (Figure 4C).

Recent studies have found that vascular growth and osteogenesis in bone are coupled with angiogenic and angiocrine factors, and ECs are highly heterogeneous and acquire special functional properties in the local microenvironment (Chim et al., 2013; Kusumbe et al., 2014; Zhu et al., 2020). In order to investigate the biological process of KD in the EC lineage, we combined all endothelial lineage cells into two samples for GO analysis and functionally analyzed GO enrichment for genes differentially upregulated in the KD samples compared with the OVCF samples. We found that the GO terms “extracellular matrix organization,” “collagen fibril organization,” “skeletal system development,” and “cell adhesion” were enriched in arterial ECs, capillary ECs, and venous ECs, especially in capillary ECs, which may contribute significantly to ossification; arterial ECs may be the cells in which the body continuously responds to the mechanical stimulus, transforming growth factor beta stimulus, and fibroblast growth factor stimulus; and venous ECs are cells with strong angiogenic and skeletal system developmental capabilities, which may be responsible for the relatively high proportion of bone cells and ECs in the KD samples (Figure 4D).

Following a preliminary understanding of the GO terms upregulated in KD by ECs, to continue to observe the heterogeneity of endothelial cell transcription factors in the KD versus the OVCF samples, we applied single-cell regulatory network inference and clustering (SCENIC) analysis (Section 2). We found that the regulation intensity of SOX17, RUNX2, and EGR1 transcription factors was upregulated in both KD and OVCF arterial ECs, of which SOX17 was required for arterial EC regeneration after inflammation-induced vascular injury (Liu et al., 2019), RUNX2 and EGR1 were both important transcription factors during osteoblast differentiation (Chiba et al., 2022;Komori, 2020), and EGR1 was slightly more regulated in KD than that in OVCF samples and was an important transcription factor mediating the response to ischemia (Yan et al., 2006) and hypoxia (Sperandio et al., 2009) (Figure 4E).

The intensity of FOXM1, PAX5, and MAFB transcription factor regulation was significantly upregulated in OVCF vein ECs. FOXM1 is a key transcription factor involved in the regulation of cell proliferation (Liao et al., 2018). PAX5 is an essential transcription factor for B-cell development and is involved in key regulatory and structural proteins in cell adhesion, migration, antigen presentation, and germinal center B-cell formation (Schebesta et al., 2007). MAFB is a transcription factor negatively regulating RANKL-induced osteoclast differentiation and acts as an important modulator of RANKL-mediated osteoclast formation (Kim et al., 2007). The AP-1 (also known as FOS) family of transcription factors (FOSB and JUN) is significantly upregulated in KD venous ECs and functions to mediate VEGF-induced EC migration and proliferation (Jia et al., 2016); FOXC1 has transcription factors that differentiate into osteoblast and chondrocyte functions (Aoki et al., 2021).

In KD and OVCF capillary ECs, the intensity of regulation of the SOX4 transcription factor is upregulated, and its function may be to induce apoptosis (David et al., 2016). However, NATC2 is significantly upregulated in KD, a member of the nuclear factor of activated T cell (NFAT) family, and it plays an important role in RANKL-induced osteoclast development (Huang et al., 2020).

Taken together, arterial ECs in KD have a strong ability to participate in arterial regeneration and promote osteoblast differentiation under inflammatory conditions, which may be the result of the body mediating ischemic and hypoxic responses, but because bone resorption is stronger than bone formation, it eventually leads to bone loss. Venous ECs have strong EC migration and angiogenesis ability and actively promote osteogenic differentiation and inhibit osteoclast differentiation, thus revealing that intraosseous vascular growth is coupled to bone formation. Significant upregulation of NATC2 transcription factors in capillary cells induced osteoclast development, but their ossification effect appeared to be strongest in GO terms, possibly as an interaction to maintain dynamic bone balance. Overall, our data reveal a high heterogeneity of ECs in human vertebral bone tissues (Figure 4E).

### 3.5 T and B cells actively participate in regulating bone homeostasis

**FIGURE 5:**
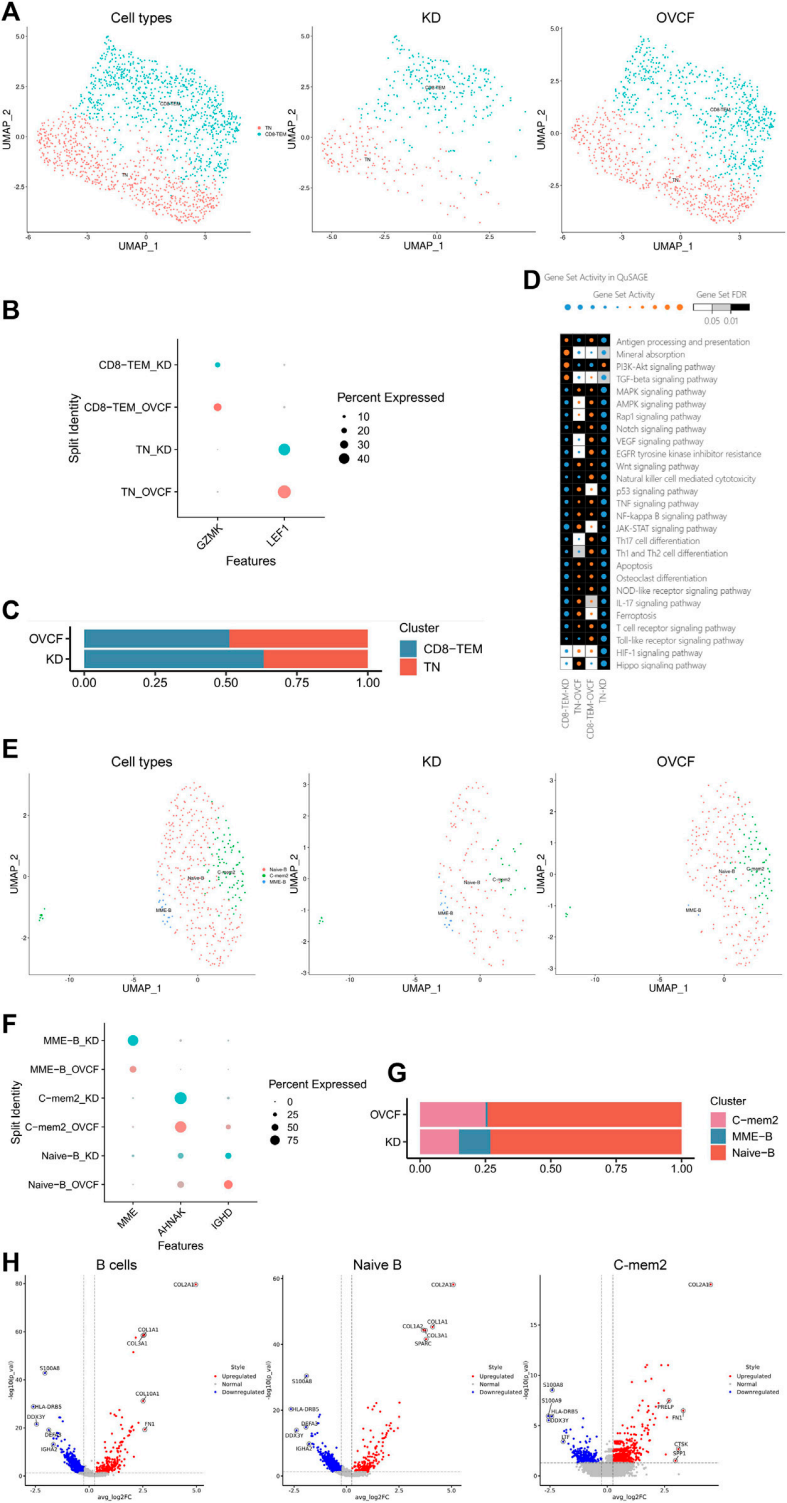
T and B cells actively participate in regulating bone homeostasis. **(A)** All T cells initially grouped were combined to reconstruct the UMAP profile, with coloration labeled by the cell type. From left to right are the KD and OVCF samples, the KD sample, and the OVCF sample. **(B)** Bubble plots of marker genes for the T-cell subcluster, with larger dots representing stronger expression and dots colored to distinguish KD and OVCF samples. **(C)** Bar chart of the T-cell subcluster in respective sample proportions. **(D)** Enriched signaling pathways were analyzed using QuSAGE analysis for two T-cell subclusters in two samples, with horizontal axis labels representing cell types in different samples, vertical axis labels representing signaling pathway gene sets, square colors representing statistical probability levels, and darker colors indicating greater significance. The orange bubble size indicates relative activation, and blue indicates relative inactivity. **(E)** All B cells initially grouped were combined to reconstruct the UMAP profile, with coloration labeled by the cell type. From left to right are KD and OVCF samples, the KD sample, and the OVCF sample. **(F)** Bubble plots of marker genes for B-cell subclusters, with larger dots representing stronger expression and dots colored to distinguish KD and OVCF samples. **(G)** Bar chart of B-cell subclusters in respective sample proportions. **(H)** Corresponding volcano plots were drawn for the top five differentially highly expressed genes up- and downregulated in the KD samples compared with the OVCF samples, with red representing differentially upregulated genes and blue representing differentially downregulated genes.

T helper cells (Th1, Th2, Treg, and Th17) and B cells are actively involved in bone homeostasis (Dar et al., 2018). In our results, after preliminary clustering, we then combined two samples of T cells for further subdivision and identification, and two cell species were preliminarily identified based on the classical marker gene reported in the literature, which were GZMK + CD8 T effector memory cells (CD8-TEM) and LEF1+T naive cells (TN) (Zhang et al., 2018). We constructed two-sample versus sub-sample UMAP plots according to the cell type (Figures 5A, B). We were surprised to find that the number of T cells in the KD samples was much less than that in the OVCF samples, with CD8-TEM versus TN accounting for 63.3% and 36.7% of the KD samples, respectively. However, CD8-TEM and TN accounted for 51.2% and 48.7%, respectively, in the OVCF samples (Figure 5C).

We applied QuSAGE analysis (gene enrichment analysis) to investigate the gene enrichment of T-cell subsets in KD versus OVCF. We found enrichment of CD8-TEM cells for “mineral absorption,” “the PI3K-Akt signaling pathway,” and “the TGF-beta signaling pathway” in KD samples, and we conclude that CD8-TEM plays an important role in regulating osteoclast bone resorption. However, the pathways enriched in both CD8-TEM and TN in OVCF were “the Wnt signaling pathway,” “osteoclast differentiation,” “the NF-kappaB signaling pathway,” and “the MAPK signaling pathway.” We concluded that T cells play an important role in regulating the above pathways and osteoblast and osteoclast differentiation during OVCFs (Figure 5D).

Finally, we combined two samples of B cells for further subdivision and identification and preliminarily identified three cell populations based on the classical marker genes reported in the literature, which were IGHD + naive B cells (naive B) (Zhang et al., 2020a) and AHNAK + classical memory B cells 2 (C-mem2). MME + B cells with high differential expression were named MME-B cells (MME + B cells, MME-B) according to their marker gene because no subtypes with consistent characteristics were found in the literature. Two-sample versus sub-sample UMAP plots were then constructed according to the cell type (Figures 5E, F). Similarly, we found that the number of B cells in the OVCF samples was almost double that in the KD samples, with C-mem2, MME-B, and naive B accounting for 14.9%, 12.1%, and 73.0% in the KD samples, respectively, and 25.1%, 0.8%, and 74.1% in the OVCF samples, and it was not difficult to find the abundance of naive B in the two samples (Figure 5G).

We mapped the top five differentially highly expressed genes upregulated and downregulated in the KD samples compared with OVCF in all B cells, naive B cells, and C-mem2 cells (Figure 5H). We were surprised to find that in C-mem2, CTSK is a classic marker of osteoclasts and plays an important role in bone resorption (Tan et al., 2021); SPP1 and FN1 are major bone matrix proteins that can be induced by Runx2 and are essential for osteoblast differentiation and chondrocyte maturation (Komori, 2019); PRELP is a heparin/heparan sulfate-binding protein, and it has been shown that the development of osteoarthritis is associated with PRELP (Lin et al., 2022), indicating that C-mem2 actively participates in bone homeostasis regulation.

### 3.6 Intercellular communication reveals the osteoimmune microenvironment in human vertebral bone tissue

**FIGURE 6:**
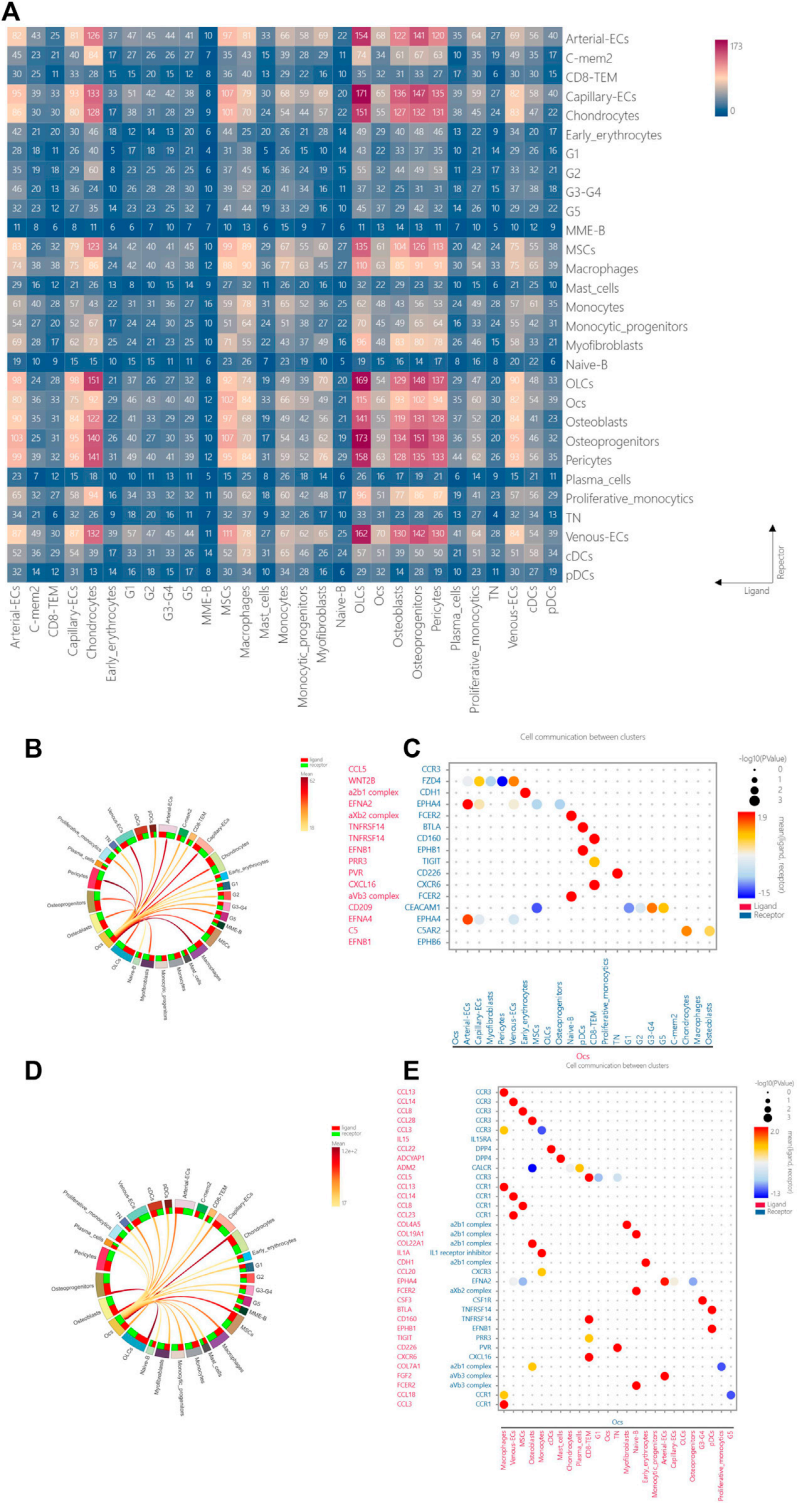
Intercellular communication reveals the osteoimmune microenvironment in human vertebral bone tissue. **(A)** CellphoneDB analysis was performed on all cell types in KD samples, with the transverse longitudinal axis as the ligand cell group and the longitudinal axis as the receptor cell group, and the colored square represents the number of protein interaction relationships between cell groups, with red representing the more significant communication and blue representing the weaker interaction. **(B)** Network relationship of osteoclasts with other cells when they act as a ligand. **(C)** Osteoclasts act as ligand–receptor pairs with other cells when they are ligands, with the transverse longitudinal axis representing cell groups with interactions, red representing ligands, blue representing receptors, the longitudinal axis representing protein interaction pairs, red representing ligands, blue representing receptors, dots representing significant sizes, larger dots representing more significance, and dot color representing the magnitude of interaction intensity, the greater the intensity, the more red, and the smaller the intensity, the more blue. **(D)** Network relationship of osteoclasts with other cells when they act as receptors. **(E)** Osteoclasts act as ligand–receptor pairs with other cells when they are receptors, with the transverse longitudinal axis representing cell groups with interaction relationships, red representing ligands, blue representing receptors, the longitudinal axis representing protein interaction pairs, red representing ligands, blue representing receptors, dots representing significant sizes, larger dots representing significant sizes, dot colors representing interaction intensity sizes, greater intensities being more biased toward red, and smaller intensities being more biased toward blue.

Elucidating the interaction between bone cells and immune cells in the osteoimmune microenvironment will help to understand the pathogenesis and potential therapeutic targets of pathological bone loss in the vertebral body and ischemic bone necrosis in the vertebral body. Because osteoclasts were not found in OVCF, to explore the osteoimmune microenvironment in KD, we utilized cell-to-cell communication analysis (CellphoneDB analysis) to analyze the cell-to-cell communication in all cell types in KD samples (Figure 6A). We found that the communication between bone cells was the most abundant, while the communication between ECs and bone cells was second only to that between bone cells, and although the communication between immune cells and bone cells was very minor, its effect in bone cells was highly significant.

In order to explore the ligand–receptor relationship between osteoclasts and immune cells, we investigated the interaction between osteoclasts and other cells. We found that osteoclasts communicate significantly with CD8-TEM, TN, arterial ECs, naive B cells, and pDCs when they act as ligands or receptors and share the same ligand–receptor/receptor–ligand pair relationship, confirming the molecular targets and potential functions of bidirectional communication between bone cells and immune cells.

When osteoclasts are used as ligands, the specific ligand pair relationship with arterial ECs is EFNA2/EFNA4–EPHA4. It should be noted that EFNA2/EFNA4 has an important regulatory role in reverse signal transduction in osteoclasts and bone resorption, while EPHA4 is considered to be an effective negative modulator of osteoclast activity (Stiffel et al., 2014). The paired relationship with naive B cells is aXb2 complex/aVb3 complex–FCER2. It should be noted that FCER2 plays an important role in regulating IgE production and B-cell differentiation, and activation of this receptor inhibits IgE production, resulting in the downregulation of IgE-mediated immune responses (Karimi et al., 2019). The paired relationship with pDCs is TNFRSF14–BTLA. Interestingly, TNFRSF14 is a membrane-bound receptor that activates the NF-kappaB signaling pathway, leading to genes that induce a pro-inflammatory response and promote cell survival (Shui et al., 2011). The TNFRSF14–BTLA cis complex competitively inhibits the activation of the ligand TNFRSF14 expressed in the surrounding microenvironment, thereby inhibiting TNFRSF14-dependent NF-kappaB activation (Cheung et al., 2009) and EFNB1–EPHB1. Consistently, type B Eph-ephrin bidirectional communication has been found to play an important role in skeletal development and skeletal repair (Arthur and Gronthos, 2021). The paired relationship with CD8-TEM is TNFRSF14–CD160. Interestingly, TNF superfamily member TNFRSF14 acts as a receptor or ligand and is involved in bidirectional cell–cell contact signaling between antigen-presenting cells and lymphocytes. The receptor on CD160 immune cells transmits stimuli or inhibitory signals that regulate cell activation and differentiation. When TNFRSF14 interacts with CD160, it provides stimulatory signals to NK cells and enhances interferon-γ(IFNG) production (Dostert et al., 2019; Sedy et al., 2014); increased binding of CXCL16–CXCR6 (CXCL16–CXCR6) can inhibit RANKL-mediated osteoclast formation, which is mainly through the inhibition of JAK2/STAT3 signaling. Thus, the CXCL16–CXCR6 axis may become a new target for therapeutic intervention in bone resorption diseases such as rheumatoid arthritis and osteoporosis (Li et al., 2012). The paired relationship with TN was PVR–CD226, and the binding of PVR–CD226 could stimulate T-cell proliferation and cytokine production, including IL2, IL5, IL10, IL13, and IFNG (Zhu et al., 2016) (Figures 6B, C).

When osteoclasts are used as receptors, there is a significant ligand pair relationship with macrophages, venous ECs, MSCs, osteoblasts, and CD8-TEM communication with C-C motif chemokine ligand family–C-C chemokine receptor family: cytokines are secreted protein families involved in immunoregulatory and inflammatory processes, of which CCR1 and CCR3 transduce signals by increasing intracellular calcium levels when used as receptors (Ponath et al., 1996). Notably, the ligand–receptor pair with osteoblasts is ADM2–CALCR (ADM2 is a member of the calcitonin hormone family and CALCR is a calcitonin high-affinity receptor, and their combination may be involved in maintaining calcium homeostasis and regulating osteoclast-mediated bone resorption (Tang et al., 2014), but this ligand–receptor pair acts significantly but less strongly in osteoclasts and osteoblasts and may be associated with reducing osteoclast-mediated bone resorption). The paired relationship with monocytes involves IL1A–IL1 receptor inhibitors. It should be noted that IL1 receptor inhibitors attenuate the activation of NF-kappaB and the three MAPK pathways— p38, p42/p44—and JNK signaling pathways by inhibiting the activity of interleukin 1α (IL1A) (Wu et al., 2004). The paired relationships with cDCs and mast cells were CCL22–DPP4 and ADCYAP1–DPP4. Notably, DPP4 can be expressed on the surface of osteoblasts, osteoclasts, and osteocytes, and its effect facilitates bone formation (Glorie et al., 2016). The matching relationship with arterial ECs involves the FGF2–aVb3 complex. Interestingly, FGF2 as a novel biomolecule could accelerate bone healing and cartilage repair after injury (Zhang et al., 2020b) (Figures 6D, E).

## 4 Discussion

Evidence of vertebral avascular osteonecrosis and pathologic bone loss remains poorly characterized during the development of OVCF to KD. Osteoimmunology emphasizes the interaction between the bone and the immune system, especially in various inflammatory bone diseases, and the development of the field of osteoimmunology has greatly promoted the study of molecular mechanisms associated with bone destruction (Dar et al., 2018). It has, therefore, become quite important to understand the interaction between bone cells and immune cells (Arron and Choi, 2000). Previous approaches to bone research have been based on whole bone cell populations rather than single cells of bone tissues, which neglect heterogeneity between single cells and lack accuracy and resolution to characterize the relationship and interaction between bone tissue cells and immune cells (Bai et al., 2019). ScRNA-seq is emerging as a popular technology in the field of bone immunology and provides unprecedented details for diseases such as osteoporosis (Wang et al., 2022), periodontitis (Chen et al., 2022), and nonunion (Avin et al., 2022). In this study, we used single-cell sequencing for the first time to analyze the molecular characteristics and evolution of OVCFs and KD and to better understand how the interaction between dysregulated bone cells and immune cells is involved in the process of vertebral ischemic osteonecrosis and pathological bone loss.

In the pathogenesis of KD, there are statements such as vertebral avascular necrosis, bone biomechanics, and gas formation, all of which have obtained clinical and biomechanical support evidence. Overall, most investigators believe that KD is the result of a combination of factors such as osteoporosis, vertebral avascular necrosis, and biomechanical changes after fracture (He et al., 2016). Among them, post-traumatic osteonecrosis is the most recognized pathogenetic hypothesis for KD (Gou et al., 2023). In our results, MSCs were found to be significantly enriched in the GO terms “response to mechanical stimulation,” “response to hypoxia,” “the interferon-γ-mediated signaling pathway,” and “response to ischemia” during differentiation. GO terms “response to hypoxia,” “response to mechanical stimulation,” and “cellular response to interferon-γ" were significantly enriched during the final differentiation into osteoclasts. Arterial ECs are significantly enriched in the GO term “response to mechanical stimulation” which is significantly upregulated in KD, while EGR1 is highly regulated in arterial ECs in KD and is an important transcription factor that mediates responses to ischemia (Yan et al., 2006) and hypoxia (Sperandio et al., 2009). The above results confirmed that the pathogenesis of KD is closely related to the involvement of MSCs, osteoclasts, and arterial ECs in the combined effects of continuous mechanical stimulation, ischemia, and hypoxia and plays a central role in the whole pathological process. More importantly, MSCs as well as osteoclasts and T cell-mediated immune responses may be important mechanisms of action in regulating bone homeostasis, especially closely related to interferon-γ. Our findings confirm that the pathological process of KD is multifactorial and complex and complements the molecular landscape and site of action of KD pathogenesis.

The current study reveals that ECs are active players in both bone formation and resorption in the skeletal system and that interdependent crosstalk of ECs and other cell populations of bone may provide new entry points for the treatment of bone erosion (Tuckermann and Adams, 2021). For example, in intercellular communication, the intercellular communication between arterial ECs and osteoclasts is significant, and the repair effect of FGF2 on bones is expected to be a new target. More importantly, according to the GO terms upregulated by ECs in KD, SCENIC analysis of the number of genes regulated by each transcription factor, and the intensity of regulation in EC subsets in the two-sample heatmap, it is shown that ECs have the functions of responding to various cytokines and recruiting immune cells, promoting skeletal system development, cell adhesion, migration, proliferation, heterogeneity, antigen presentation, and immune enhancement. ECs have been suggested to be novel immune cells with many innate immune functions, which have also been demonstrated (Chen et al., 2022; Shao et al., 2020). Our data also showed that ECs and bone cells were enriched in the KD samples, while immune cells were significantly reduced compared with the OVCF samples. These results indicate that in the relative absence of immune cells in the KD samples, ECs actively exert immunomodulatory functions, thereby participating in inflammatory responses and promoting osteogenesis. However, it has been studied that osteogenesis is coupled to vascular growth under normal expression, but vascular endothelial growth factor (VEGF) overexpression instead leads to impaired osteogenesis (Burkhardt et al., 1987), while no strong evidence seems to be found in our results, which still needs to be confirmed by further studies in the future. Interestingly, pericytes in KD outnumber those in OVCFs by 20 times, which might indicate an unidentified differential role of pericytes in bone development and repair under these conditions (Zhu et al., 2022).

The tumor necrosis factor superfamily (TNFSF) and its corresponding receptor superfamily (TNFRSF) form communication pathways required for *in vivo* developmental, homeostatic, and stimulatory response processes. In particular, it plays a specific role in immune system function. CD160 is a glycosyl phosphoinositide (GPI)-linked receptor that is expressed at low levels in many cells but shows high expression in NK cells. The interaction of TNFRSF14 with CD160 opens up unique immunological properties for the TNFR signaling pathway (Sedy et al., 2014). We were surprised to find that interferon-γ production by T cells strongly inhibited osteoclastogenesis by interfering with the RANKL ± RANK signaling pathway (Hu et al., 2018), while in cell-to-cell communication, we found that osteoclasts communicated significantly with CD8-TEM and enhanced IFNG production when CD160 interacted with TNFRSF14. We conclude that osteoclasts and T cells inhibit osteoclastogenesis by interacting with TNFRSF14 through CD160, which may be a positive feedback regulatory mechanism. In addition, osteoclasts inhibited osteoclastogenesis when interacting with ligand–receptor pairs for pDCs, CD8-TEM, and TN (TNFRSF14–BTLA, CXCL16–CXCR6, PVR–CD226), and monocytes with ligand–receptor pairs for osteoclasts (IL1A–IL1 receptor inhibitors). However, both cDCs and mast cells interacting with the ligand–receptor pairs of osteoclasts (CCL22–DPP4 and ADCYAP1–DPP4) and osteoclasts interacting with the ligand–receptor pairs of pDCs (EFNB1–EPHB1) have effects on bone development and bone repair and favor bone formation. According to the results of intercellular communication, inhibition of osteoclast bone resorption and promotion of bone formation will become important topics, and the sites of action of the above immune cells (pDCs, cDCs, CD8-TEM, TN, mast cells, and monocytes) and osteoclasts are expected to be new therapeutic targets. In bone loss diseases, such as rheumatoid arthritis, immune mechanisms of joint damage and interactions between the immune system, synovial fibroblasts, and bone will help identify new therapeutic targets for RA (Komatsu and Takayanagi, 2022). Therefore, understanding the crosstalk between immune cells and bone cells has a broad therapeutic prospect, and more and more in-depth bone immunological studies are still needed to confirm it in the future and will bring new therapeutic targets for bone loss diseases.

In conclusion, we used scRNA-seq to comprehensively characterize the osteoimmune microenvironment with multiple cell types and molecular mechanisms in human vertebral bone tissues. This study identifies alterations in osteoimmunology cell types that occur in the context of OVCFs versus KD, including MSCs, fibroblast subsets, monocyte subsets, EC subsets, T cells, and B cells. These results will enable us to identify changes between OVCFs and KD in a cell type-specific manner in further investigations. This may accelerate mechanistic and functional studies of osteoimmune cell types and specific gene action in vertebral avascular necrosis and pathological bone loss diseases, paving the way for drug discovery.

## Data Availability

The data presented in the study are deposited in the Gene Expression Omnibus (GEO) repository, accession number GSE242414 (https://www.ncbi.nlm.nih.gov/geo/, GSE242414).
